# Long-term exposure to a 40-GHz electromagnetic field does not affect genotoxicity or heat shock protein expression in HCE-T or SRA01/04 cells

**DOI:** 10.1093/jrr/rrz017

**Published:** 2019-06-14

**Authors:** Shin Koyama, Eijiro Narita, Yukihisa Suzuki, Takeo Shiina, Masao Taki, Naoki Shinohara, Junji Miyakoshi

**Affiliations:** 1 Laboratory of Applied Radio Engineering for Humanosphere, Research Institute for Sustainable Humanosphere, Kyoto University, Uji, Kyoto, Japan; 2 Department of Electrical & Electronic Engineering, Graduate Schools of Science and Engineering, Tokyo Metropolitan University, 1-1, Hachioji, Tokyo, Japan

**Keywords:** millimeter waves, micronucleus formation, comet assay, heat shock protein, human eye cells

## Abstract

Millimeter waves are used in various fields, and the risks of this wavelength range for human health must be carefully evaluated. In this study, we investigated the effects of millimeter waves on genotoxicity and heat shock protein expression in human corneal epithelial (HCE-T) and human lens epithelial (SRA01/04) cells. We exposed the cells to 40-GHz millimeter waves at 1 mW/cm^2^ for 24 h. We observed no statistically significant increase in the micronucleus (MN) frequency or the level of DNA strand breaks in cells exposed to 40-GHz millimeter waves relative to sham-exposed and incubator controls. Heat shock protein (Hsp) expression also exhibited no statistically significant response to the 40-GHz exposure. These results indicate that exposure to 40 GHz millimeter waves under these conditions has little or no effect on MN formation, DNA strand breaks, or Hsp expression in HCE-T or SRA01/04 cells.

## INTRODUCTION

New communications technologies have developed rapidly over the past few decades. In particular, wireless communication has become ubiquitous throughout the world. This rapid change is unlikely to stop, even if it is revealed to have negative consequences. The use of various wavelengths of the electromagnetic spectrum results in depletion of available radio frequencies, creating demand for access to new frequency ranges. The rapid introduction of wireless devices contributes to anxiety about human health. Recently, millimeter-wave [30–300 gigahertz (GHz)] technologies have been developed, and will be used for the next generation of Ultra-Broadband in 5 G cellular networks. Hence, we sought to investigate the effects of millimeter waves on human epithelial cells. Previously, we studied the effect of long-term exposure to 60-GHz millimeter-wave radiation on micronucleus (MN) formation, DNA strand breaks, and heat shock protein (Hsp) expression in cells derived from the human eye [1]. We sought to evaluate the effects of genotoxicity using MN formation and comet assays. In addition, we sought to assess physiological effects by monitoring Hsp expression. For this purpose, we studied three Hsps: Hsp27, Hsp70 and Hsp90α [2]. Hsp27 is a chaperone protein that belongs to the small heat shock protein family, which also includes ubiquitin and α-crystallin. Because we used eye cells in this study, we suspected that α-crystallin was related to Hsp expression. Hsp70 is the most widely studied Hsp, and is typically expressed during stresses such as heat shock and UV irradiation. Hsp90 is another extensively studied Hsp that assists other proteins to fold properly, stabilizes proteins against heat stress, and aids in protein degradation. These proteins might be involved in mediating the effects of exposure to electromagnetic radiation. In that study, we detected no obvious adverse effects of 60-GHz exposure. However several other studies reported some non-thermal effects on biological organization [3–6]. In 2013, the International Agency for Research on Cancer (IARC) classified radiofrequency into Group 2B, ‘possibly carcinogenic to humans’ [7]. A recent study [8] indicated that co-exposure to 60.4-GHz millimeter waves and 2-deoxyglucose affected several genes associated with transcriptional repression, cellular communication, and endoplasmic reticulum homeostasis. In addition, Belpomme *et al.* indicated in their review that many types of paper showed non-thermal adverse effects of electromagnetic fields [9]. On the other hand, several studies reported no physiological effects of millimeter-wave exposure [10–12]. Thus, the effects of millimeter-waves exposure on biological systems remain controversial. Accordingly, it is necessary to evaluate the influence of millimeter waves on the human body. Because the energy of millimeter waves is absorbed by the body surface, the skin and the eye are the main tissues of concern [13, 14]. To investigate the non-thermal effects of this type of radiation, we manufactured a device that can expose cells to 40-GHz millimeter waves. This study is almost the same as the previous study except for the frequency of the waves being investigated. The 40-GHz millimeter waves will be used for train radio communication systems or public image transfer systems differently from 60 GHz. In addition, 40 GHz will be used for high-speed wireless access networks in combination with 60 GHz. It is necessary to investigate possible non-thermal effects of each frequency, because non-thermal effects are generally considered to be frequency dependent. In this study, we assessed MN formation, DNA breaks, and Hsp expression in human eye epithelial cells exposed to 40-GHz millimeter waves.

## MATERIALS AND METHODS

### Millimeter-wave exposure set-up

An applicator based on a printed circuit board (PCB) was used to expose cells to 40-GHz millimeter waves. The concept of the applicator has been described in detail in another paper [15], in which 60-GHz millimeter waves were used. The applicator used in this experiment was of the same design, except that the frequency was 40 GHz. Due to the difference in the wavelength, the dimensions of the applicators differed by a factor of ~1.5.

**Fig. 1.**
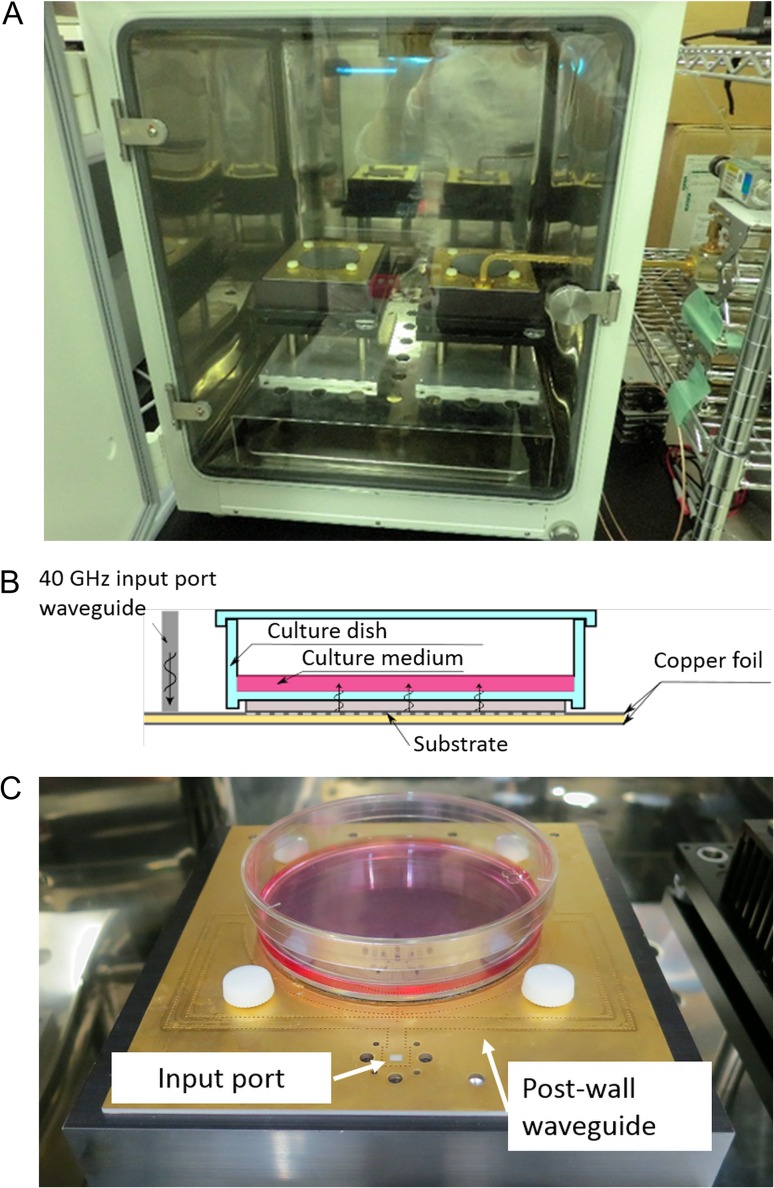
The incubator for exposure equipped with applicators for exposure (right) and sham-exposure (left) (A). Illustration of the structure (B) and the photograph (C) of the applicator.

Figure 1A is a photograph of the incubator for the 40-GHz millimeter-wave exposure. The chamber of the incubator was maintained under controlled conditions in an atmosphere of 95% air containing 5% CO_2_ at a relative humidity of >95% and a temperature of 37°C. The applicators for exposure (right) and for sham-exposure (left) were equipped in the incubator. Figure 1B illustrates the structure of the applicator, and Fig. 1C is a photograph of the applicator.

The applicator was made using a PCB with a disc-shaped area surrounded by a post-wall waveguide to feed millimeter waves into the substrate of the disc-shaped area, on which a culture dish could be placed. A culture dish 100 mm in diameter was positioned on the disc-shaped area. The 40-GHz millimeter waves were applied from the bottom through coupling windows of fine slots opened in the top metal layer of the PCB. Thus, adherent cells on the bottom of the culture dish were exposed to millimeter waves. Temperature elevation was suppressed to <0.1°C using a heat sink unit located beneath the PCB.

The spatially averaged power density was set at 1 mW/cm^2^ at the bottom of culture medium. The distribution of the power density was estimated numerically, and 61% and 82% of the area of the bottom layer of the culture dish was exposed at the power density within an average ±3 dB and ±5 dB, respectively [16].

### Cell culture

HCE-T cells [17] (RIKEN CELL BANK, Tsukuba, Japan) derived from human corneal epithelia were maintained in DMEM + HamF12 (1:1) medium supplemented with 5% fetal bovine serum (FBS), 5 μg/ml insulin and 10 ng/ml human epidermal growth factor. SRA01/04 cells (RIKEN CELL BANK) derived from human lens epithelia were maintained in DMEM medium supplemented with 20% FBS. This cell line was kindly supplied by Dr Hiroshi Sasaki of Kanazawa Medical University. Twenty-four hours after millimeter-wave exposure, the cells were harvested. The average power density was 1 mW/cm^2^ in the medium. As positive controls, the cells were treated with 10 μg/ml bleomycin (Wako Pure Chemical Industries, Ltd, Osaka, Japan) for 1 h (for the genotoxicity test) or heat treatment (for the Hsp expression test).

### Micronucleus frequency

The methodology for the micronucleus (MN) formation test was described previously [18]. Briefly, after millimeter-wave exposure or 10 μg/ml bleomycin treatment for 1 h, the cells were cultured in medium supplemented with 3 μg/ml cytochalasin B (Sigma-Aldrich, Tokyo, Japan) in a conventional incubator for 24 h, centrifuged onto slides (Shandon Southern Instruments, Cambridge, UK) at 100 × *g* for 5 min in a Cytospin centrifuge, fixed with cold 80% ethanol for 30 min, and stained with 0.2 μg/ml propidium iodide (Sigma-Aldrich). In a total of 300 binucleated cells, MN formation was counted according to modified criteria described previously [18], using a fluorescence microscope (Olympus, Tokyo, Japan). We modified the number of counting binucleated cells, because the ratios of the MN frequency were the same as in previous data. At least three independent tests were performed in all experiments.

### Comet assay

The comet assay was performed to detect DNA strand breaks at the single-cell level; the methodology was described previously [19]. Briefly, cells were exposed to millimeter-wave radiation for 24 h, collected by trypsinization and centrifugation immediately after exposure, and mixed with low–melting point agarose to prepare a cell suspension in 0.1% agarose/phosphate-buffered saline (PBS). After gelation of the agarose, the cells were lysed. For the alkaline comet assay, which detects single-strand breaks, the resultant DNA samples were electrophoresed at 1 V/cm for 30 min in 0.3 M NaOH and 1 mM ethylenediamine-N,N,N’,N’-tetraacetic acid solution. After the DNA was stained with SYBR Green I, immunofluorescence images were captured on a fluorescence microscope. DNA strand breaks were analyzed using the Comet software (Perceptive Instruments, Suffolk, UK). At least 100 comets from each gel were analyzed. Tail length indicates the pixel length of the comet tail. Tail percentage indicates the percentage of tail content relative to comet content. Tail moment was calculated as follows:

(1) $$\begin{array}{c} \mathrm{Tail moment}=\\ \left(\mathrm{the distance between the center of the comet head and the center of the comet tail}\right)\\ \times \left(\mathrm{Tail percentage}\right)/\mathrm{100} \end{array}$$

### Hsp expression

The methodology for measuring Hsp expression was described previously [19]. Briefly, after millimeter-wave exposure or heat treatment, the cells were washed with cold PBS and collected using a cell scraper. Proteins were extracted with CelLytic™-M (Sigma-Aldrich) supplemented with protease inhibitor cocktail (Sigma-Aldrich). Extracted proteins were incubated at 4°C for 15 min, and then centrifuged at 100 × *g* for 15 min. Supernatants were collected, and the concentrations were measured using a calibration curve on an iMark plate reader (Bio-Rad, Hercules, CA, USA); protein concentrations were adjusted to 1 mg/ml in all samples. The samples were then mixed with 2ME sample buffer (Wako Pure Chemical Industries) at a ratio of 1:1, incubated at 100°C for 1 min, and immediately placed on ice. Twenty micrograms of extracted protein was loaded onto 12.5% sodium dodecyl sulfate (SDS)-polyacrylamide gel (Wako Pure Chemical Industries), separated by electrophoresis, and transferred to nitrocellulose membrane (Life Technologies Japan, Tokyo, Japan) by iBlot (Life Technologies Japan). All samples (three controls, three shams, three exposures and three positive controls) in three experiments were loaded on one gel. After blotting, the BenchPro™ 4100 (Invitrogen, CA, USA) was used for the blocking and immunostaining. Membranes were blocked with 1.5% skim milk (DS Farma Biomedical, Osaka, Japan) for 1 h, and then immunostained with antibodies for 1 h. Primary antibodies were obtained from the indicated suppliers: Hsp27 (R&D Systems, MN, USA), Hsp70 (StressMarq Biosciences, BC, Canada), Hsp90α (StressMarq) and β-actin (Sigma-Aldrich). Secondary antibodies were anti-mouse (GE Healthcare, Tokyo, Japan), anti-goat (R&D), and anti-rabbit (Sigma-Aldrich). After immunostaining, membranes were stained with horseradish peroxidase, and then analyzed using the ATTO Image Analysis Software (Tokyo, Japan).

### Statistical analysis

The data were analyzed using Dunnett’s test. A *P*-value of <0.05 or <0.01 was considered to be statistically significant.

## RESULTS

### Frequency of MN formation

**Fig. 2.**
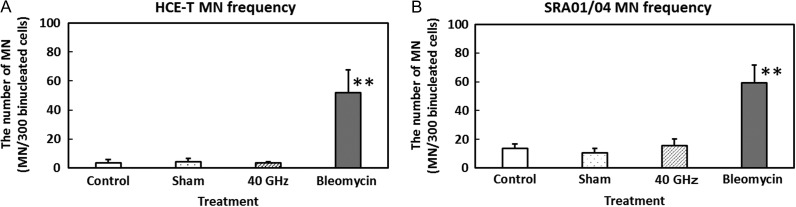
Micronucleus frequency in cells exposed to 40-GHz millimeter-wave radiation for 24 h: HCE-T cells (A) and SRA01/04 cells (B). The positive control was treatment with bleomycin (10 μg/ml). Data are presented as means ± SD from six independent experiments. **P < 0.01.

The MN frequencies in HCE-T and SRA01/04 cells are shown in Fig. 2A and B, respectively. MN frequencies in both cell types increased significantly following bleomycin treatment, used as a positive control, but no significant difference was observed between incubator control, sham exposure, and 40-GHz millimeter wave–exposed cells. These results suggest that 24-h exposure to 40-GHz millimeter-wave radiation has no significant effect on MN frequency in HCE-T or SRA01/04 cells.

### Comet assay

**Fig. 3.**
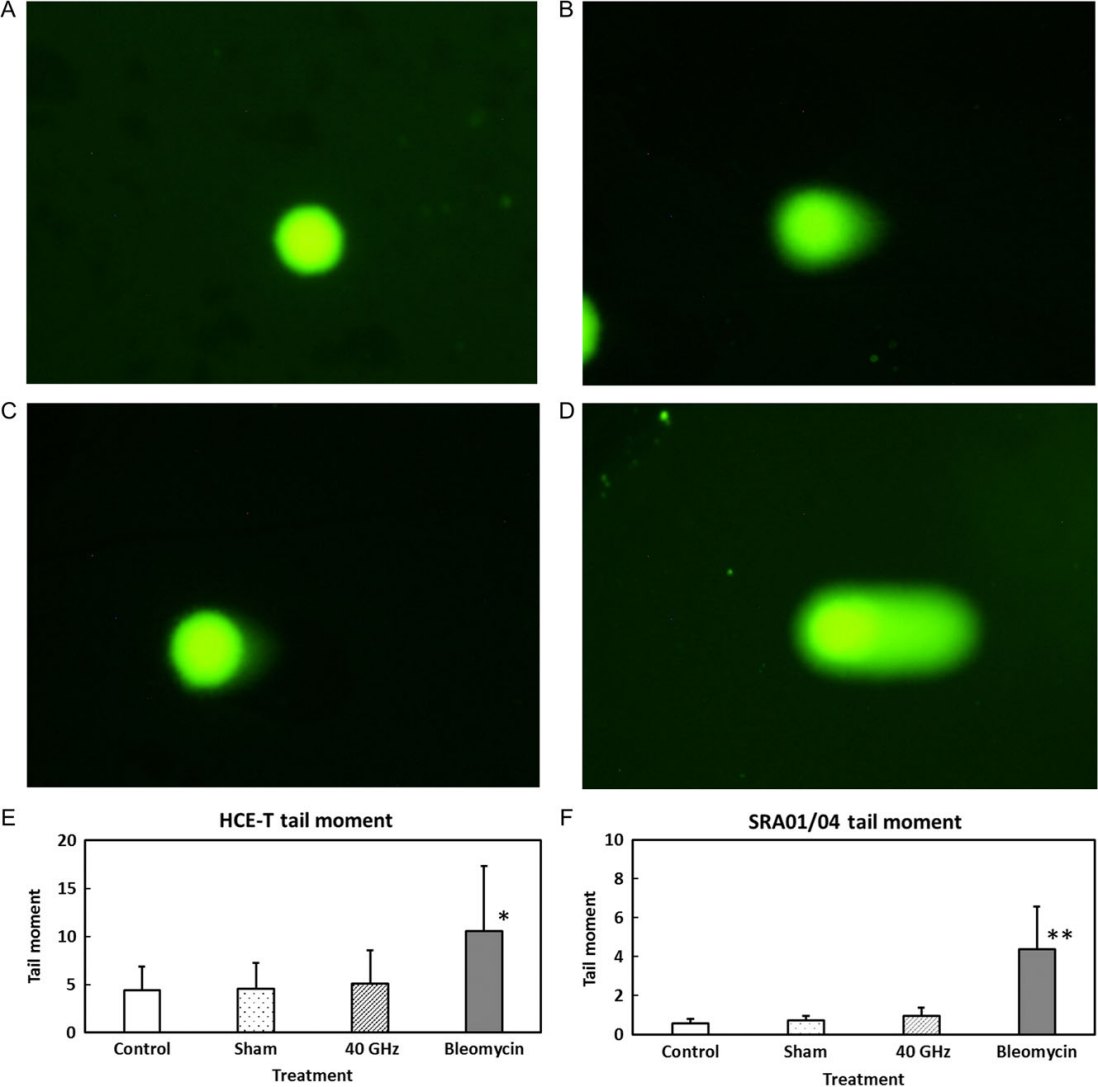
Representative micrographic photographs of control (A), sham (B), 40 GHz-exposed (C) and bleomycin-treated cells (D). Values of a comet parameter (tail moment) for cells exposed to 40-GHz millimeter-wave radiation for 24 h: HCE-T (E) and SRA01/04 (F). The positive control was treatment with bleomycin (10 μg/ml). Data are presented as means ± SD from six independent experiments. One asterisk and two asterisks indicate *P* < 0.05 and < 0.01, respectively.

Two photographs in Fig. 3A, B, C and D indicate the representative samples of control, sham, 40 GHz-exposed and bleomycin-treated cells in the comet assay, respectively. The tail moments of lysed HCE-T and SRA01/04 cells are shown in Fig. 3E and F, respectively. The tail moment indicates the severity of the genotoxic effect on the DNA. The tail moments of both HCE-T and SRA01/04 cells increased significantly following bleomycin treatment, used as a positive control, but no significant difference in tail moment was observed between the incubator control, sham exposure, or 40-GHz millimeter wave–exposed cells. These results suggest that 24-h exposure to 40-GHz millimeter-wave radiation has no significant effect on DNA breaks in HCE-T or SRA01/04 cells.

### Hsp expression

**Fig. 4.**
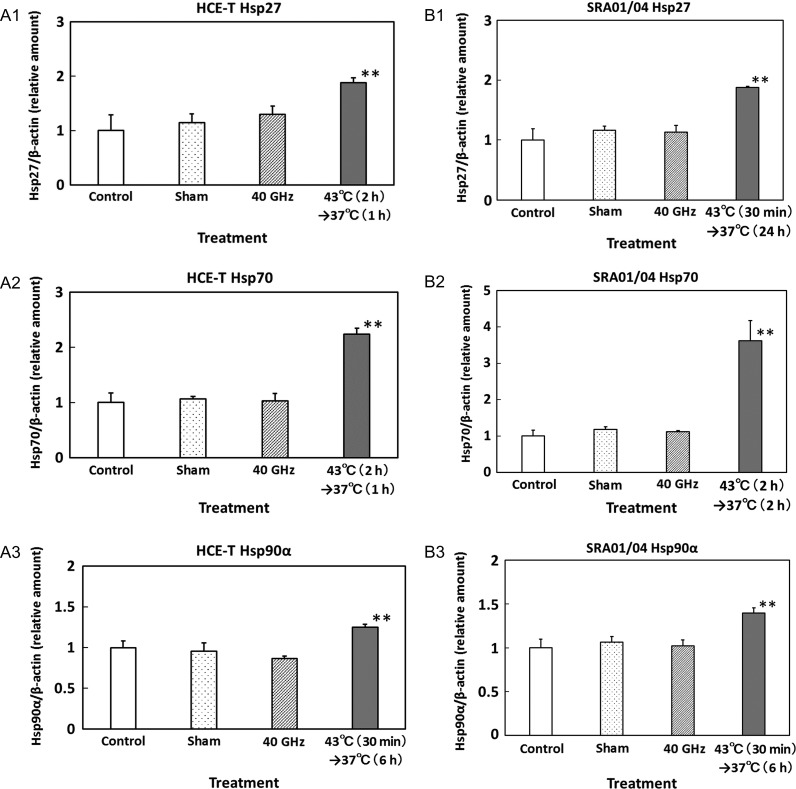
(A) Expression of Hsp27 (1), Hsp70 (2), and Hsp90α (3) in HCE-T cells exposed to 40-GHz radiation for 24 h. Positive controls underwent heat treatment, as shown in the graphs. Data are presented as means ± SD from three independent experiments. **P < 0.01. (B) Expression of Hsp27 (1), Hsp70 (2) and Hsp90α (3) in SRA01/04 cells exposed to 40-GHz radiation for 24 h. Positive controls underwent heat treatment, as shown in the graphs. Data are presented as means ± SD from three independent experiments. **P < 0.01.

The expression levels of Hsp27, 70 and 90α in HCE-T cells are shown in Fig. 4A-1, -2 and -3, respectively. Heat treatment [Hsp27 and 70: 43°C (2 h) and 37°C (1 h); Hsp90α: 43°C (30 min) and 37°C (6 h)] increased the level of all Hsps. The expression levels of Hsp27, 70 and 90α in SRA01/04 cells are shown in Fig. 4B-1, -2 and -3, respectively. Heat treatment [Hsp27 and Hsp90α: 43°C (30 min) and 37°C (24 h); Hsp70: 43°C (2 h) and 37°C (2 h)] significantly increased the level of all Hsps. However, no increase in Hsp expression was observed in the 40-GHz millimeter wave–exposed cells. These results suggest that 24-h exposure to 40-GHz millimeter-wave radiation had no significant effect on the expression of Hsp27, 70 or 90α in HCE-T or SRA01/04 cells.

## DISCUSSION

Previously, we investigated the effects of exposure to 60-GHz millimeter-wave radiation, at a constant temperature (37°C) for 24 h at the average power density of 1 mW/cm^2^, on cellular genotoxicity and stress responses in HCE-T and SRA01/04 cells [1]. HCE-T or SRA01/04 cells are derived from human cornea or lens, respectively. The penetration depth of millimeter-waves is not very deep on a human body, so we targeted the surface, and in particular human eyes, which are very sensitive compared with skin. We observed no effects on MN formation, single-strand breaks in DNA, or expression of Hsp 27, 70 or 90α. In this study, we investigated the effects of 40-GHz millimeter-wave radiation at 1 mW/cm^2^ for 24 h under the same conditions. The results for each parameter were almost the same as in the previous study.

Over the past decade, millimeter-wave radiation has spread widely due to the development of technologies that use these wavelengths. However, the biological effects of millimeter-wave exposure have not been well studied. To date, several studies have examined the biological effects of millimeter-wave exposure on neural activity [20], cell proliferation [21], cell metabolism [22], and genomic instability [4]. Haas *et al.* recently reported that 60.4-GHz millimeter-wave exposure has no effect on protein expression of Hsp70 or certain membrane receptors [23]. Although they did observe a slight increase in protein expression, they attributed it to a thermal effect. We also observed that 60-GHz millimeter-wave exposure did not increase Hsp70 expression. In addition, we have studied the biological effects of long-term exposure to 60.4-GHz millimeter waves; we found that these conditions exert very little if any genotoxicity in human eye epithelial cells [1]. Other studies obtained similar results. Haas *et al.* also reported that 24-h exposure to 60-GHz millimeter waves has no impact on dopamine turnover or dopamine transporter protein expression in NGF-treated PC12 cells, although they did observe a slight (but insignificant) increase in the level of 3,4-dihydroxyphenylacetic acid [24]. The same group observed no effect of 60.4-GHz exposure on the keratinocyte transcriptome [8], although they did see a slight alteration in the transcriptome following co-exposure to millimeter waves and 2-deoxyglucose. Yaekashiwa *et al.* reported no non-thermal effects following exposure of human skin fibroblast and glioblastoma cells to 70–300 GHz using a tunable system [25]. In summary, although several studies have examined the biological effects of millimeter-wave exposure, no study has yet obtained positive results.

In this study, we investigated the genotoxicity of 40-GHz millimeter waves in human eye epithelial cells. Very few studies have examined the effect of this wavelength on this type of cells, but some positive data have been obtained in cells exposed to similar frequencies. Recently, Vlasova *et al.* indicated that 32.9–39.6-GHz exposure enhanced the neutrophil response to particulate agonists [26]. The frequency used in that experiment was similar to the one we used, although power density was almost 10-fold higher, and this difference alone might have affected the results. Some positive results have been obtained when 42.2-GHz millimeter waves were used on murine cells or mice in *in vivo* experiments [27–32]. However, these experiments used conditions totally different from ours. The mechanisms underlying these positive data remain unclear, and they may be related to thermal effects.

The results of this study suggest that exposure of eye epithelial cells to 40-GHz millimeter-wave radiation has little or no effect on genotoxicity or protein expression. These results were consistent with our previous data obtained with 60-GHz millimeter irradiation. Although several previous studies reported some effects of these wavelengths, the mechanisms remain unclear. In future work, we should perform further experiments for cells derived from other organs, and we will proceed to clarify the effects of millimeter waves using other, highly sensitive detectors, i.e. RNA-sequencing, time-lapse microscopy, etc., because slight biological changes that cannot be detected by existing technologies might be expressed by the millimeter exposure.
